# The impact of community nucleic acid testing on infection in residential compounds during a city-wide lockdown

**DOI:** 10.1038/s41598-023-48585-5

**Published:** 2023-12-04

**Authors:** Zhenzhen Jia, Jianqiang Hu, Teng Lian, Lixian Qian, Wen Yu, Cheng Zhang

**Affiliations:** 1 School of Management, Xi’an Jiaotong University, Xi’an, 710049 China; 2 School of Management, Fudan University, Shanghai, 200433 China; 3 International Business School Suzhou, Xi’an Jiaotong-Liverpool University, Suzhou, 215123 China

**Keywords:** Epidemiology, Quality of life

## Abstract

The question of whether community nucleic acid testing contributes to an increase in infections within residential compounds has not been definitively answered. Shanghai, one of the largest cities in China, conducted city-wide community testing during its lockdown from late March to May 2022. This situation provided a unique opportunity to examine the effect of community testing on infection rates, as the lockdown largely eliminated confounding factors such as citizen mobility. In our study, based on a survey of 208 residential compounds in Shanghai and the daily infection data during the lockdown period, we found a significant correlation between community testing and infection risk in these compounds. However, after addressing potential issues of reverse causality and sampling bias, we found no significant causal link between community testing and infection risk. Furthermore, we discovered that increased awareness of mask-wearing correlated with a decrease in infections within the residential compounds during community testing. This suggests that the perceived correlation between community testing and infection risk may be confounded by residents’ adherence to mask-wearing practices. Our findings emphasize the need for public health decision-makers to reinforce the importance of mask-wearing during community testing, as a means to prevent infections among citizens.

## Introduction

Large-scale nucleic acid testing has proven pivotal in promptly identifying infected individuals during the COVID-19 outbreak in China^1^. Although the nucleic acid test is the most effective method for detecting infections, its efficiency is significantly constrained by the limited medical testing resources available for adequately covering wide geographical areas^2^. To circumvent this, governments worldwide, including those in the United States, China, and South Korea, began implementing centralized nucleic acid testing, or community testing, from 2020 onwards, pooling resources to test specific regional populations within a short timeframe^3^. As a strategy to combat further virus spread, from 2021, the Chinese government routinized centralized testing as a regular measure at the residential compound level. This involved organizing residents within each compound to undergo multiple rounds of compulsory centralized nucleic acid tests^1^. By pooling time and location, centralized testing at the residential compound level (hereafter referred to as community testing) is expected to be more efficient than traditional decentralized methods. This approach has been deployed in several Chinese cities, including Xi’an, Jilin, Shenzhen, Shanghai, and Beijing, to curb the rapid spread of the Delta and Omicron variants. The Chinese Center for Disease Control and Prevention has stated that community testing during a COVID-19 outbreak is an efficient measure for identifying infections^1^. In Hong Kong, community testing is also believed to effectively identify undetected infections and transmission chains^4^.

During the Omicron outbreak in Shanghai from March to May 2022, community testing emerged as a key mechanism for discovering new cases. Testing was predominantly organized within residential compounds, particularly during the city-wide lockdown. Testing procedures were conducted outdoors, with strict adherence to social distancing and mask-wearing protocols. The frequency of testing was determined by the demands of epidemic prevention and control, with compounds reporting higher infection numbers receiving more frequent tests. Factors such as district GDP level and the availability of medical resources also influenced the frequency of testing. During the lockdown period, the cost of nucleic acid testing was covered by the local government^5^, and medical professionals from all over China were mobilized to Shanghai to supplement local healthcare resources for nucleic acid testing^6^.

However, alongside the potential benefits of community testing, concerns have been raised about the increased risk of infections among those queuing for tests^3^. This issue came into sharp focus during the 2022 Omicron surge in Shanghai, where infection numbers in residential compounds continued to rise despite several weeks of city-wide lockdown. If community testing does indeed increase infection risk, policymakers must seriously reconsider the trade-off between testing efficiency and infection risk^7^. Many attribute the ongoing spread to community testing, given its requirement for residents to congregate within compounds. In an online survey conducted as part of our study, nearly 97% of Shanghai participants believed that queuing for community testing could increase infection risk. Even after the Shanghai government pointed to several possible sources of infection during the lockdown period (e.g. virus-contaminated food, undetected infected family members, and non-compliance with personal protection), there remains a lack of comprehensive data and rigorous statistical analysis on this issue. To the best of our knowledge, no study has directly addressed this matter by providing a definitive answer based on statistical evidence.

Our study exploited the lockdown period from late March to May 2022 in Shanghai to assess the infection risk from community testing, as the lockdown measures largely precluded other infectious factors such as resident mobility between and within residential compounds. The study, based on a survey of 208 residential compounds in Shanghai and daily infection data during the lockdown, found a significant correlation between community testing and infection risk. However, after accounting for potential reverse causality and sampling bias, we found no significant causal link between the two. Interestingly, within the same economic analysis framework, we discovered a negative correlation between mask-wearing awareness and infection rates. This suggests that frequent community testing does not significantly increase infection risk, provided residents adhere to standardized self-protective measures like mask-wearing.

## Method

### Data preparation

We have two data sources: the residential addresses of newly-infected patients published every day on the Shanghai government Wechat account and the responses to our questionnaire. Based on the residential addresses of the infected patients published on the “Shanghai Release” public accounts, we obtain the number of days with newly-infected residents per week in each residential compound during the Omicron outbreak period, which captures the infection situation of each residential compound. From May 4 to May 10, 2022, we conducted a questionnaire survey of all Shanghai residents who were on lockdown against the epidemic at that time. This survey was approved by the Institutional Review Board of School of Management, Fudan University and was performed in accordance with relevant guidelines and regulations. Informed consent was obtained from all participants and their privacy was guaranteed throughout the study. We received a total of 278 responses (representing 208 unique residential compounds) to the questionnaire, with respondents from 14 of 16 administrative districts in Shanghai, all except for Chongming and Jinshan. In addition to the situation of community testing, the questionnaire also investigated other factors that may accelerate or suppress the spread of the epidemic, which can help us accurately analyze the impact of community testing on the spread of the epidemic.

To strengthen the rigor of our research, we conduct the following data elimination. First, we reject the data that was not filled in completely or seriously, leaving 215 valid responses. Second, we remove the records filled by participants who had already been infected during the broader COVID-19 epidemic because the number of nucleic acid tests of these people may be different from the average number of nucleic acid tests in the residential compound. After these eliminations, there remain 208 respondents. Because our study is conducted on the residential compound level, we combine the data of participants living in the same residential compound; our data thus include 175 unique residential addresses.

### Variable construction

#### Dependent variable—The degree of infection

We use the number of days per week that every residential compound had newly-infected residents to measure the degree of infection. Considering that there may be a lag in the release of infection data, we introduce different lags (i.e., 1 to 7 days) when calculating the number of days with newly-infected residents per week based on the government’s announcements of the residential addresses with new infections. The greater the number of days with newly-infected residents per week in the residential compound, the more serious the infection situation is. The number of newly-infected patients per week in the residential compound would be the most direct indicator; however, this data is not publicly available for our study.

#### Independent variable —The degree of community testing

We measure the degree of community testing using the number of nucleic acid tests per week in each residential compound. Our data encompass the number of nucleic acid tests across all residential compounds over a 6-week period.

#### Control variables

We measure several residential compound factors that may affect the spread of infection: the size and disinfection situation of the residential compound, and the mobility and protective behaviors of residents. First, we use the number of buildings and the number of floors in each building to measure the size of the residential compound. The number of buildings has three levels: less than 10 buildings, 10 to 20 buildings, and more than 20 buildings. The number of floors is divided into two levels: 1 to 6 floors and more than 6 floors. Buildings with more than 6 floors are required to install elevators. Second, we measure the disinfection situation of the residential compound from two perspectives, the weekly disinfection frequency and the disinfection level for express delivery parcels. Third, we use the residents’ range of activities during the epidemic and the range of express delivery parcels to measure the mobility of residents in the residential compound. Regarding the residents’ range of activities, we use 5 levels to describe the mobility of residents, ranging from quarantined at home to free movement in the residential compound. Regarding the range of express delivery parcels, we use 5 levels to describe the mobility of residents, from self-pickup at the gate of the residential compound to volunteers delivering directly to the doorstep. Lastly, we measure the protective behavior of residents during the epidemic based on their level of compliance with social distancing and their level of awareness regarding mask-wearing. The compliance level with social distancing includes three levels: “a small number of residents comply, and most do not”, “most residents comply, and a small number do not”, and “all residents comply”. The awareness of mask-wearing is gauged by the proportion of residents who wear masks properly during community testing in the residential compound. It is categorized into three levels: “a small number of residents wear masks properly, and most do not”, “most residents wear masks properly, and a small number do not”, and “all residents wear masks properly”. Table 1 presents the summary statistics for these characteristics of residential compounds (See the Supplement File for the survey questions on residential compound factors used in this study).

**Table 1:** Summary statistics for the characteristics of residential compounds.

Characteristics of residential compounds		Summary statistics		
Size	Number of buildings (%)	< 10 (15.91%)	[10, 20) (17.42%)	$\ge$ 20 (66.67%)
	Number of floors (%)	[1 - 6] (46.21%)	> 6 (53.79%)	

		Mean	Standard deviation	Range ([min–max])
Disinfection situation	Weekly disinfection frequency	2.076	1.893	[0-4]
	Disinfection level for express delivery parcels	2.303	0.751	[0-3]
Mobility	Residents’ range of activities	2.864	0.963	[0-4]
	Range of express delivery parcels	2.076	1.137	[0-4]
Protective behavior	Compliance with social distancing	1.182	0.675	[0-2]
	Awareness of mask-wearing	1.682	0.468	[0-2]

Apart from the aforementioned factors related to the residential compound, we also consider the number of households, the number of residents, and the age and gender distribution of residents (sourced from the seventh census data) within the district where the residential compound is located. These factors help describe the residential density information of the compound, which may influence the degree of infection within the compound. Ideally, we would refine the data to specific residential areas, but we were unable to locate relevant information.

In addition, we also measure two time-series factors related to the degree of infection: the number of days with newly-infected residents in the residential compound during the previous week and the number of newly-infected residents in the district where the residential compound is located during the previous week. The greater the number of days with newly-infected residents during the previous week, the greater the number of days with newly-infected residents there may be in the following week because of the contagiousness and incubation period of the virus. Similarly, the greater the number of newly-infected residents in the district where the residential compound is located during the previous week, the more severe the outbreak in the district, and the greater the number of days with newly-infected residents there may be in the following week.

### Model

To investigate the impact of community testing on the degree of infection, we first establish a Poisson log-linear regression model. This model, a form of generalized linear model used to model count data^8^, is suitable for our research given the count data characteristics of infection degree. In this model, we analyze the relationship between $I_{i, w}$, which denotes the number of days with newly-infected residents in residential compound *i* during week *w*, and $S_{i, w}$, which indicates the number of nucleic acid tests administered in residential compound *i* during week *w*. The control variables $C_{i}$ as introduced in Table 1 are included in the model to control the factors that may also impact the infection condition of the residential compound. $B_{c}$ represents the set of coefficients corresponding to control variables $C_{i}$. The coefficient of interest is $\beta _{s}$. The mathematical specification for this model is:

1 $$\begin{aligned} ln({\lambda ^I_{i, w}}) = \beta _{0} + \beta _{s} S_{i, w} + B_{c}C_{i}, \end{aligned}$$

where $\lambda ^I_{i, w}$ stands for the conditional mean of $I_{i, w}$ given $S_{i, w}$ and $C_{i}$. The results of Model (1) can only illustrate the correlation between community testing and the degree of infection, but cannot explain the causal relationship between the two. For example, if the correlation between the two is positive, we cannot determine whether the increase in community testing resulted in more infection or the increase in the degree of infection led to more community testing organized by the government (reverse causality).

To address the reverse causality concern in estimating the model, we use the common approach of an instrumental variable (IV)^9^. A valid IV needs to have an impact on the endogenous variable and impact the dependent variable ($I_{i, w}$) only through the endogenous variable ($S_{i, w}$). We use whether the average GDP level of the district where the residential compound is located is higher than the mean value as an IV, $G_{i}$, given that the cost of nucleic acid testing in the context of epidemic lockdown is primarily covered by local governments and funded through local finances^5^. Model (2) can be used to formulate the IV approach and estimate the impact of the degree of community testing on the degree of infection:

2 $$\begin{aligned} \begin{aligned} ln({\lambda ^I_{i, w}}) = \beta _{0} + \beta _{s} S_{i, w} + B_{c}^{1}C_{i},\\ ln({\lambda ^S_{i, w}}) = \beta _{1} + \beta _{g} G_{i} + B_{c}^{1}C_{i}, \end{aligned} \end{aligned}$$

where $\lambda ^S_{i, w}$ stands for the conditional mean of $S_{i, w}$ given $G_{i}$ and $C_{i}$. We apply a two-stage estimation procedure. Specifically, in the first stage, the second regression equation in model (2) is fitted to obtain the estimated values of $S_{i,w}$’s. Then, in the second stage, these estimated values are substituted for $S_{i,w}$’s in the first regression equation to estimate the coefficients.

## Results

### The positive correlation between community testing and infection risk

#### Correlation analysis

Without any control, the degree of community testing has a positive correlation with the degree of infection. The correlation between the weekly count of days with new infections among residents – lagged by 0 to 7 days – and the weekly number of nucleic acid tests is sequentially 0.177, 0.155, 0.139, 0.128, 0.114, 0.097, 0.080, and 0.070. Each correlation is significant at the 0.01 level.

#### Regression analysis

We conduct a further Poisson regression estimation controlling for residential compound-level covariates, including the size and disinfection situation of the residential compound, the mobility and protective behaviors of residents, residential density, and temporal effects (ref. Model (1)). The results show consistent positive correlations between community testing and infection risk with different time lags (ref. Table 2).

**Table 2:** Results of Model (1).

	Number of days with newly-infected residents per week							
	Lag 0	Lag 1	Lag 2	Lag 3	Lag 4	Lag 5	Lag 6	Lag 7
Number of community testing per week	0.020$$^{***}$$ (0.004)	0.021$$^{***}$$ (0.004)	0.019$$^{***}$$ (0.004)	0.019$$^{***}$$ (0.004)	0.019$$^{***}$$ (0.004)	0.018$$^{***}$$ (0.004)	0.018$$^{***}$$ (0.004)	0.018$$^{***}$$ (0.004)
Awareness of mask-wearing	$$-$$0.238$$^{***}$$ (0.066)	$$-$$0.237$$^{***}$$ (0.066)	$$-$$0.224$$^{***}$$ (0.066)	$$-$$0.230$$^{***}$$ (0.066)	$$-$$0.237$$^{***}$$ (0.066)	$$-$$0.235$$^{***}$$ (0.066)	$$-$$0.250$$^{***}$$ (0.066)	$$-$$0.242$$^{***}$$ (0.066)
Other controls	$\checkmark$	$\checkmark$	$\checkmark$	$\checkmark$	$\checkmark$	$\checkmark$	$\checkmark$	$\checkmark$
Observations	1225	1225	1225	1225	1225	1225	1225	1225
Log likelihood	$-$1677.346	$-$1681.666	$-$1665.558	$-$1672.241	$-$1671.309	$-$1682.116	$-$1674.779	$-$1667.837

$$^{***}$$ Indicates significance level of 0.01.

### Community testing does not increase infection risk

One major concern about a seemingly significant positive correlation is reverse causality^10^. In this case, increasing infections in the residential compound may cause the government to organize more nucleic acid tests in response. Our findings resolve this issue, showing that the positive correlation between community testing and infection risk is specious.

Our study applies an IV approach^9^ to resolve the reverse causality concern. IV is an additional variable that should satisfy two conditions: (1) it should have an impact on the endogenous variable (i.e. community testing in this case), and (2) it should not have an impact on the outcome variable (i.e. infection risk in this case) other than through the endogenous variable. In the presence of the effect of IVs, if the relationship between the independent and outcome variables remains, then it is more likely that there is a hypothesized causal relationship between the two^11^. In this context, our study uses the average GDP level of each residential compound’s district as an IV. More specifically, we use a relative measure of this IV, i.e. whether the regional GDP is higher or lower than the mean value. This variable satisfies the requirement of a valid IV: (1) financial income is one major decision-making factor in the local government’s plan for the frequency of community testing; and (b) GDP is exogenous to the infection risk, meaning that a local government’s previous financial income does not affect regional infection risk directly. This IV also passes the weak IV test^12^, suggesting that this IV has a strong correlation with the endogenous variable. A two-stage estimation technique is then applied to test the regression model with the IV (ref. Model (2)). All covariates remain the same as in the previous regression analysis.

The results are provided in Table 3, revealing the insignificant effect of community testing on infection risk in various model settings. This implies that the previous significant positive correlation between the two is superficial and misunderstood, and that it may actually indicate reverse causality.

**Table 3:** Results of Model (2).

	Number of days with newly-infected residents per week							
	Lag 0	Lag 1	Lag 2	Lag 3	Lag 4	Lag 5	Lag 6	Lag 7
Number of community testing per week	$-$0.004 (0.017)	$-$0.006 (0.018)	$-$0.003 (0.017)	$-$0.006 (0.017)	$-$0.012 (0.017)	$-$0.008 (0.017)	$-$0.009 (0.017)	$-$0.007 (0.017)
Awareness of mask-wearing	$$-$$0.245$$^{***}$$ (0.066)	$$-$$0.246$$^{***}$$ (0.066)	$$-$$0.232$$^{***}$$ (0.066)	$$-$$0.238$$^{***}$$ (0.066)	$$-$$0.247$$^{***}$$ (0.066)	$$-$$0.243$$^{***}$$ (0.066)	$$-$$0.257$$^{***}$$ (0.066)	$$-$$0.248$$^{***}$$ (0.066)
Other controls	$\checkmark$	$\checkmark$	$\checkmark$	$\checkmark$	$\checkmark$	$\checkmark$	$\checkmark$	$\checkmark$
Observations	1225	1225	1225	1225	1225	1225	1225	1225
Log likelihood	$-$1676.308	$-$1680.493	$-$1664.663	$-$1671.112	$-$1669.606	$-$1680.880	$-$1673.390	$-$1666.714

$$^{***}$$ Indicates significance level of 0.01.

### The negative correlation between the degree of mask-wearing and infection risk

If community testing does not really affect infection risk, what factors may actually affect infection risk during testing? As public health literature suggests, social distancing, that is, engaging in interactions safely and at a distance (e.g. wearing masks), is one effective measure to slow the spread of COVID-19^13–15^. As community testing may reduce the physical distance among residents (e.g. when they are crowded in the testing queue), whether residents wear masks and keep appropriate social distance may affect the infection risk. To evaluate the possible effect of such precautions, we measure the proportion of residents wearing masks in the residential compound during community testing. This is treated as a function of residents’ awareness of mask regulations, as introduced in the "Method" section. The stronger the residents’ awareness of mask-wearing regulations, the more likely it is that more residents will be wearing masks.

To test the effect of mask-wearing, we follow the same regression model with the IV (ref. Model (2)). The results in Table 3 show that the coefficients of mask-wearing are significantly negative, suggesting that the stronger the residents’ awareness of mask regulations, the lighter the infection risk in the residential compound.

## Discussion

To investigate the robustness of our results, we apply propensity score matching^16^, a method designed to address the issue of sampling bias. In our context, residential compounds with different degrees of community testing may have differences in residential compound-related characteristics, including the size and disinfection situation of the residential compound and the mobility and protective behaviors of residents. These differences may lead to differences in the infection situation of residential compounds with different degrees of community testing. Hence, it is hard to say that a statistically significant difference in the infection situation in residential compounds is caused by the degree of community testing rather than other confounding factors. Propensity score methods provide the tools to design and analyze observational data in which the treatment is not randomly assigned so observations in different treatment groups may systematically differ from each other. To conduct propensity score matching, we divide the number of nucleic acid tests in a certain week into two levels: *high* and *low*. If the number of nucleic acid tests in a given week for a residential compound exceeds the average frequency for all residential compounds that week, we label this residential compound as having a *high* degree of community testing for the corresponding week; otherwise, it falls under a *low* degree of community testing. Through propensity score matching, we create balanced samples where residential compounds with *high* and *low* degrees of community testing have no significant differences with respect to all control variables (ref. Table 1). Then we run the regression model with the IV (ref. Model (2)) on the balanced samples to illustrate the causal relationship between community testing and infection risk.

**Figure 1:**
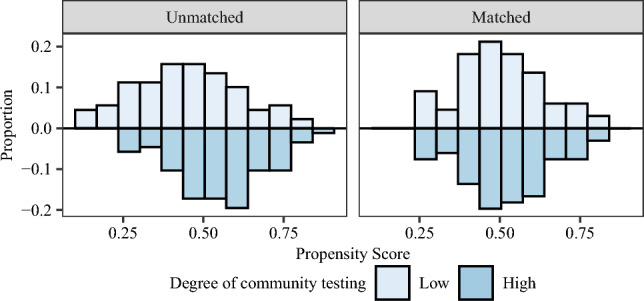
Propensity score distribution before and after matching.

After matching, the standardized differences in the means of the covariates in the treated and control groups, defined as the difference in means for each covariate as a percentage of its standard deviation from the treated group^16^, are reduced to 0.2, satisfying the reasonable balance criteria suggested in the literature^17,18^. Figure 1 visualizes the change, showing significant differences before matching, while after matching, both treated and control groups are indistinguishable in the propensity score distribution.

Regression results with the IV on the matched samples (see Table 4) reveal an insignificant relationship between community testing and infection risk, while demonstrating a significant negative relationship between mask-wearing awareness and infection risk.

**Table 4:** Results of Model (2) on matched samples.

	Number of days with newly-infected residents per week							
	Lag 0	Lag 1	Lag 2	Lag 3	Lag 4	Lag 5	Lag 6	Lag 7
Number of community testing per week	0.0004 (0.026)	0.006 (0.026)	0.005 (0.026)	0.003 (0.026)	$-$0.007 (0.026)	$-$0.003 (0.025)	$-$0.004 (0.025)	$-$0.003 (0.026)
Awareness of mask$$-$$wearing	$$-$$0.275$$^{***}$$ (0.085)	$$-$$0.277$$^{***}$$ (0.084)	$$-$$0.249$$^{***}$$ (0.085)	$$-$$0.262$$^{***}$$ (0.085)	$$-$$0.283$$^{***}$$ (0.085)	$$-$$0.274$$^{***}$$ (0.084)	$$-$$0.283$$^{***}$$ (0.084)	$$-$$0.277$$^{***}$$ (0.085)
Other controls	$\checkmark$	$\checkmark$	$\checkmark$	$\checkmark$	$\checkmark$	$\checkmark$	$\checkmark$	$\checkmark$
Observations	917	917	917	917	917	917	917	917
Log likelihood	$-$1007.620	$-$1019.713	$-$1000.727	$-$1004.678	$-$1006.028	$-$1010.772	$-$1002.901	$-$1004.552

$$^{***}$$ Indicates significance level of 0.01.

## Conclusion

Although community nucleic acid testing (compared to decentralized and casual nucleic acid testing) helps public health authorities trace regional outbreaks in a shorter period of time and thus designate appropriate intervention policies more quickly, public health authorities are also concerned about whether centralized testing causes the virus transmission that may lead to further outbreaks. This is an ongoing concern for people in epidemic outbreak areas, especially since the end of 2019 as the COVID-19 epidemic has spread worldwide. For large-scale outbreaks, China adopted the lockdown strategy and conducted community testing to detect infected people as soon as possible. However, many people have raised the question whether community testing in residential compounds leads to more infections in the compounds and thereby delays the control of the COVID-19 epidemic.

Our study is the first to investigate whether community testing increases infection risk and thus epidemic spread. We find no statistical evidence to support that community testing increases the infection risk of the Omicron variant outbreak. In addition, we find that the stronger the residents’ awareness of wearing masks, the lighter the Omicron epidemic in the residential compound. Therefore, standardized epidemic prevention measures can effectively reduce infection risk. The results of our study give people who are fighting against the epidemic reassurance – as long as people observe effective personal protection measures, there is no need to take a negative attitude toward community testing.

In future research, we can consider more detailed indicators to measure the infection status and include more characteristic factors of the residential compounds in our research. First, it would be more reasonable to use the number of infected patients to measure the infection status of the residential compound, but we could not obtain this information from the released epidemic data, which is a limitation of our study. Second, we also hope to conduct further analysis in combination with other factors such as the age of the residential compound and the resident composition to improve our understanding of the heterogeneous effect of infections in the residential compound.

### Supplementary Information

Supplementary Information.

## Data Availability

The datasets generated and/or analyzed during the current study are not publicly available due to the sensitivity of the data, but are available from the corresponding author on reasonable request.
