# Supplementary material for: The impact of community nucleic acid testing on infection in residential compounds during a city-wide lockdown
Source: Sci Rep. 2023 Dec 4;13:21334. doi: 10.1038/s41598-023-48585-5 (PMC10696007; doi:10.1038/s41598-023-48585-5)
Supplement: Supplementary file 1 — Supplementary Information. [file 41598_2023_48585_MOESM1_ESM.pdf]

The Impact of Community Nucleic Acid Testing on Infection in Residential Compounds during a City-wide Lockdown

Zhenzhen Jia[1], Jianqiang Hu[2,*], Teng Lian[2], Lixian Qian[3], Wen Yu[2], and Cheng Zhang[2]

[1]Xi'an Jiaotong University, School of Management, Xi'an, 710049, China
[2]Fudan University, School of Management, Shanghai, 200433, China
[3]Xi'an Jiaotong-Liverpool University, International Business School Suzhou, Suzhou, 215123, China
[*]hujq@fudan.edu.cn

Appendix: Survey questions on residential compound factors

1. Number of buildings

   How many buildings are in your residential compound?

   - Less than 10.
   - 10 to 20.
   - More than 20.

2. Number of floors

   How many floors are there in each building in your residential compound?

   - 1 to 6.
   - More than 6.

3. Weekly disinfection frequency

   What is the weekly disinfection frequency in your residential compound?

   - More than 6 times.
   - 3-6 times.
   - Less than 3 times.
   - No disinfection.
   - Do not know.

4. Disinfection level for express delivery parcels

   Does your residential compound disinfect express delivery parcels during the lockdown period?

   - During the entire lockdown period, the residential compound always disinfected express delivery parcels.
   - After the initial lockdown period, the residential compound began to disinfect express delivery parcels.
   - During the entire lockdown period, the residential compound did not disinfect express delivery parcels.
   - Other.

5. Residents' range of activities

   Which of the following measures have been used in your residential compound during the lockdown period? (multiple choices)

   - All residents are completely free to move in the residential compound.
   - Residents of the buildings with infected residents are quarantined at home, other residents are free to move in the residential compound.

- All residents are quarantined at home.
- Other.

6. Range of express delivery parcels

   Which of the following express delivery methods have been used in your residential compound during the lockdown period? (multiple choices)

   - All residents pick up their own parcels at the gate of the residential compound.
   - For residents of the buildings with infected residents, volunteers deliver parcels directly to their doorstep, and other residents pick up their parcels at the gate of the residential compound.
   - For residents of the buildings with infected residents, volunteers deliver parcels to the gate of the building, and other residents pick up their parcels at the gate of the residential compound.
   - Volunteers deliver parcels to the gate of the building.
   - Volunteers deliver parcels directly to the doorstep.

7. Compliance with social distancing

   Do residents follow the rule of keeping a distance of two-meter while doing nucleic acid testing in the residential compound?

   - All residents do.
   - Most residents do, and a small number of residents do not.
   - A small number of residents do, and most residents do not.

8. Awareness of mask-wearing

   Do residents wear masks in your residential compound?

   - All residents wear masks.
   - Most residents wear masks, and a small number of residents do not wear masks.
   - A small number of residents wear masks, and most residents do not wear masks.
